# Microbial Removal of Petroleum Hydrocarbons from Contaminated Soil under Arsenic Stress

**DOI:** 10.3390/toxics11020143

**Published:** 2023-02-01

**Authors:** Qu Su, Jiang Yu, Kaiqin Fang, Panyue Dong, Zheyong Li, Wuzhu Zhang, Manxia Liu, Luojing Xiang, Junxiong Cai

**Affiliations:** 1 Hubei Provincial Academy of Eco-EnvironmentSal Science, Wuhan 430070, China; 2 State Environmental Protection Key Laboratory of Soil Health and Green Remediation, Wuhan 430070, China; 3 Institute of Advanced Studies, China University of Geosciences, Wuhan 430079, China; 4 Huazhong Agricultural University, Wuhan 430070, China

**Keywords:** bioremediation, heavy metal stress, petroleum hydrocarbon removal, soil pollution

## Abstract

The contamination of soils with petroleum and its derivatives is a longstanding, widespread, and worsening environmental issue. However, efforts to remediate petroleum hydrocarbon-polluted soils often neglect or overlook the interference of heavy metals that often co-contaminate these soils and occur in petroleum itself. Here, we identified *Acinetobacter* baumannii strain JYZ-03 according to its Gram staining, oxidase reaction, biochemical tests, and FAME and *16S* rDNA gene sequence analyses and determined that it has the ability to degrade petroleum hydrocarbons. It was isolated from soil contaminated by both heavy metals and petroleum hydrocarbons. Strain JYZ-03 utilized diesel oil, long-chain n-alkanes, branched alkanes, and polycyclic aromatic hydrocarbons (PAHs) as its sole carbon sources. It degraded 93.29% of the diesel oil burden in 7 days. It also had a high tolerance to heavy metal stress caused by arsenic (As). Its petroleum hydrocarbon degradation efficiency remained constant over the 0–300 mg/L As(V) range. Its optimal growth conditions were pH 7.0 and 25–30 °C, respectively, and its growth was not inhibited even by 3.0% (*w*/*v*) NaCl. Strain JYZ-03 effectively bioremediates petroleum hydrocarbon-contaminated soil in the presence of As stress. Therefore, strain JYZ-03 may be of high value in petroleum- and heavy-metal-contaminated site bioremediation.

## 1. Introduction

Alkanes, aromatic compounds, nitrogen, and sulfur-oxygen-containing compounds are the major constituents of total petroleum hydrocarbons (TPHs). The aromatic fraction comprises compounds with benzene rings and includes polycyclic aromatic hydrocarbons (PAHs). These latter contain multiple fused aromatic rings and are listed as priority pollutants, as they are carcinogenic, mutagenic, toxic, and environmentally recalcitrant [1,2]. The development of land that has been polluted by oil refineries and other chemical industries is highly problematic. Prior research on the disposal or reclamation of these contaminated soils has ignored the interference of heavy metals. However, many oil-polluted sites are co-contaminated with heavy metals [3]. In earlier studies, petroleum hydrocarbons and heavy metals were detected in contaminated soils near gas stations, automobile repair workshops, and power stations. In fact, crude oil already contains heavy metals. Osuji [4] performed a qualitative analysis of the ash content in crude oil from domestic and foreign oilfields and detected Zn, Cu, Pb, Cd, Ni, Mn, Co, and 27 other metals. These elements and hydrocarbons constitute petroleum components and may interact with each other [5]. Heavy metal pollution may also be introduced to soils via the drilling fluid additives and low-quality barite used during extraction and mining [6]. Common hazardous metals such as Zn, Pb, Cu, Cd, Ni, Hg, Ba, and Cr were detected in at least trace amounts in all drilling waste tested [7]. Giller [8] and Olaniran [9] reported that heavy metals have different toxic effects on microorganisms and the environment as they vary in terms of bonding and mobility. Each heavy metal species can occur in the form of colloids or soluble complexes differing in toxicity and substrate competition. The physicochemical properties of heavy metals and petroleum hydrocarbons in the soil influence the environmental behavior of the pollutants and affect their transmembrane transport and biodegradation.

Heavy metals have complex effects on the soil environment. The efficiency of microbial petroleum hydrocarbon biodegradation tends to decrease with increasing heavy metal concentration [10,11,12]. Few studies have investigated microbial co-removal of petroleum hydrocarbon and heavy metals. Nevertheless, *Pseudarthrobacter phenanthrenivorans*, *Sphingomonas paucimobilis*, *Citrobacter freundii*, *Stenotrophomonas maltophilia,* and other species induce defense systems in response to heavy metal stress [13,14,15]. Oriomah [11] reported that *Achromobacter xylosoxidans* separately tolerated Cu (II) and waste oil. When both contaminants coexisted, however, the ability of *A. xylosoxidans* to degrade waste oil decreased from 40% to 5% after the Cu (II) concentration increased to 200 mg/L. Baltrons [16] discovered that inhibition of microbial degradation of 3–4-ring PAHs increased with heavy metal concentration. Heavy metals inhibit bacterial petroleum hydrocarbon biodegradation by damaging cells and/or reducing their viability [17]. Heavy metals also affect the functional properties of biologically active substances such as cellular enzymes and proteins. However, certain enzymes and organic acids may intracellularly and extracellularly reduce and/or complex heavy metals [18,19]. As these defense responses may occupy or even monopolize the normal metabolic pathways of bacterial cells, they may also bypass or deviate from petroleum hydrocarbon metabolism [20,21]. Heavy metals can also affect bacterial metabolic pathways by inhibiting petroleum hydrocarbon-degrading enzymes. Liu [22] found that exposure to 100–300 mg/L Pb (II) altered the spatial conformation of *Bacillus malacitensis* catechol 2,3-dioxygenase. The catalytic active site of the enzyme was blocked, reactive oxygen species (ROS) were induced, and the enzyme protein was damaged. Earlier studies have largely ignored or neglected the impact of heavy metals on petroleum-contaminated sites. Bioremediation research has focused almost exclusively on biological oil removal without regarding the potential interference of heavy metals in this process. For these reasons, the mechanisms of petroleum hydrocarbon removal by soil microorganisms under heavy metal stress must be elucidated.

## 2. Materials and Methods

### 2.1. Sample Collection

A contaminated soil sample was collected from the soil surface (0–20 cm) of a plot near a gas station in Wuhan, Hubei Province, China. The soil sample was placed in cloth bags, transferred to a laboratory, and stored at −20 °C until the subsequent analyses. Certain soil samples were stored at 4 °C until microbial screening and petroleum hydrocarbon content determination. The remaining soil was air-dried and passed through a 2 mm mesh sieve to remove large particles. Selected physicochemical properties of the soil were then determined (Table 1).

### 2.2. Media

Hydrocarbon-degrading bacteria were isolated in mineral salt medium (MSM) consisting of 1.0 g NH_4_NO_3_, 1.0 g NaCl, 0.2 g MgSO_4_ 7H_2_O, 7.5 g K_2_HPO_4_, 2.0 g KH_2_PO_4_, and 1% (*w*/*v*) diesel oil in 1 L pure water at pH 7.0−7.2.

The Luria-Bertani (LB) medium consisted of 10 g NaCl, 10 g tryptone, 5 g yeast extract, and 1 L pure water at pH 7.2–7.4. The beef extract-peptone medium consisted of 5 g NaCl, 10 g peptone, 3 g beef extract, and 1 L pure water at pH 7.0–7.2.

### 2.3. Enrichment and Isolation of Petroleum Hydrocarbon-Degrading Bacteria

A total of 5 g of contaminated soil sample was blended with 20 sterilized glass beads, and the mixture was added to 95 mL sterilized ultrapure water, shocked, and incubated at 150 rpm and 30 °C for 4 h. The suspension stood 10 min, and the bacteria-laden supernatant was collected. The first enrichment was performed by inoculating 5 mL soil suspension into 45 mL MSM containing 100 mg/L diesel oil as the sole carbon source. The suspension was cultured on a shaker table for 7 days and transferred to fresh MSM every 7 days. Bacterial growth was monitored daily by the optical density at 600 nm (OD_600_). When the medium became turbid or discolored, it was diluted with a concentration gradient, coated with beef extract peptone medium, and incubated at 30 °C for 1–2 days. Single colonies were purified to isolate those derived from a single strain. All isolates were as stored at −80 °C in the form of liquid cultures containing 20% sterilized glycerol.

### 2.4. Petroleum Hydrocarbon Degradation Assay

The petroleum hydrocarbon-degrading bacteria were cultured in LB medium until the middle of the logarithmic growth phase and centrifuged in a 50 mL centrifuge tube at 8000× *g* rpm for 5 min. The supernatant was discarded, and the bacteria were suspended in sterile physiological saline. The preceding operations were repeated thrice. The OD_600_ of the final bacterial suspension was adjusted to 1.2, and it was inoculated into fresh MSM with different environmental factors. The efficiency of strain JYZ-03 at degrading the various components in diesel oil was investigated. The effect of varying the NaCl concentration (0.1, 0.5, 1.0, 2.0, or 3.0% as follows: add 5.85 g, 27.9 g, 55.8 g, 111.6 g, or 167.4 g NaCl to 100 mL MSM, respectively), the initial seeding dose (1.0, 2.0, 3.0, 4.0, or 5.0%), the pH (5.0, 6.0, 7.0, 8.0, or 9.0), and the temperature (20, 25, 30, 35, or 40 °C) of the culture medium were also investigated.

All biodegradation experiments were performed in MSM containing diesel oil as the sole carbon and energy source. The flasks were incubated at 150 rpm for 7 days, and bacterial growth was monitored daily by measuring the increases in culture OD_600_. After 7 days of incubation, any remaining petroleum hydrocarbon was extracted with n-hexane (1:1 *v*/*v*). One milliliter organic phase was passed through an organic filter membrane and then analyzed by gas chromatography (GC) in flame ionization detection (FID) mode. The gas chromatograph used in the test was a GC2010-Plus model with a quartz capillary column type SH-Rtx-1 (30 m × 0.25 mm × 0.25 μm). The operating parameters were as follows: split ratio of 33.81:1; air flow rate: 400 mL/min; hydrogen flow rate: 40 mL/min; carrier gas (nitrogen) flow rate: 3.0 mL/min; makeup gas flow rate: 30 mL/min; injection volume: 1 μL; injection port temperature: 300 °C; FID detector temperature: 325 °C; temperature program: increase to 230 °C at a rate of 40 °C/min, increase to 320 °C at a rate of 20 °C/min, and hold for 20 min.

The degradation rate was estimated by calculating the GC profile of the petroleum hydrocarbon substrate. The strain cell growth was evaluated by measuring the increase in culture OD_600_.

The degradation rate was calculated as follows:

(1) $$\mathrm{Degradation}\left(\%\right)=\frac{a-b}{a}\times 100$$

where a is the mass of the petroleum hydrocarbon in the control and b is the mass of the petroleum hydrocarbon remaining after treatment.

### 2.5. Carbon Source Utilization

The strain JYZ-03 was also tested for its ability to grow on and degrade 500 mg/L long-chain alkanes (n-hexadecane, n-triacontane, n-dotriacontane, n-tetratriacontane, n-hexatriacontane, and n-octatriacontane) and 50 mg/L polycyclic aromatic hydrocarbons (PAHs; naphthalene and phenanthrene) in MSM as sole carbon source, respectively. All chemicals were of analytical grade and obtained from Sigma-Aldrich Corp., St. Louis, MO, USA. The foregoing extraction and degradation efficiency measurement methods in 2.4 were applied to the long-chain alkanes. The PAHs were extracted with 1:1 (*v*/*v*) acetone:n-hexane. Bacterial growth was monitored by measuring the increase in culture OD_600_.

### 2.6. Biochemical Test

The shape, Gram staining, spore formation, and colony morphology of the bacteria growing on a solid LB medium were examined by transmission electron microscopy (TEM). Biochemical reactions, contact enzyme, methyl red (M-R), and emulsification properties were also determined.

### 2.7. Effects of Heavy Metal Stress on Bacterial Growth and Petroleum Hydrocarbon Degradation

A JYZ-03 suspension (OD_600_ = 0.8–1.0; 2% *v*/*v*) was added to LB medium containing 0, 20, 50, 100, 150, 200, 250, 300 mg/L As(V) or Pb^2+^ to explore the inhibitory effect of heavy metals on strain JYZ-03 growth. The flasks were incubated at 30 °C, and 150 rpm for 12 h, and the OD_600_ were measured and cultured under the same conditions for 7 days to investigate the effects of various As(V) and Pb^2+^ concentrations in MSM on the petroleum hydrocarbon degradation efficiency of strain JYZ-03. Petroleum hydrocarbons were added at a 1:1 ratio of diesel volume to culture medium volume.

### 2.8. Identification of the Selected Bacterial Isolates

The genomic DNA of the bacterial isolates was extracted by standard methods, and 16S rRNA gene sequencing was performed by the dideoxy sanger method. The 16S rRNA gene was amplified with universal bacterial primers in a thermal cycler. The reaction was conducted in a total volume of 25 mL. The PCR program was 10 min at 95 °C, 35 cycles of 30 s at 94 °C, 30 s at 52–65 °C, and 90 s at 72 °C. The sequences were compared against available nucleotide sequences deposited in the National Center for Biotechnology Information (NCBI) database (https://www.ncbi.nlm.nih.gov/nuccore (accessed on 7 September 2022)) by using the Basic Local Alignment Search Tool (BLAST; https://blast.ncbi.nlm.nih.gov/Blast.cgi (accessed on 7 September 2022)). Phylogenetic trees were plotted by the neighbor-joining (NJ) method using MEGA X software (https://www.megasoftware.net/dload_win_gui (accessed on 8 September 2022)). The sequences of the selected strains were submitted to the National Center for Biotechnology Information (NCBI) GenBank database (https://www.ncbi.nlm.nih.gov/genbank/ (accessed on 8 September 2022)) and assigned accession numbers.

### 2.9. Evaluation of the Efficiency of TPH from Soil by JYZ03

The actual soil collected was air-dried, ground through 2 mm sieve and mixed with diesel fuel, and aged for 10 d away from light to make the petroleum hydrocarbons distributed evenly in the soil. The bacteria were incubated in LB medium with strain JYZ-03 for 24 h, centrifuged at 8000× *g* rpm for 5 min, washed with sterile water 3 times, and adjusted the bacterial suspension OD_600_ = 0.9. Three groups were designed, petroleum hydrocarbon single contamination group, petroleum hydrocarbon-arsenic composite group, and control group; each group was set up in three parallel.

Petroleum hydrocarbon single pollution group: the initial concentration of petroleum hydrocarbon was 4103.90 mg/kg, and 10 mL of bacterial suspension of degradation bacteria was inserted. Petroleum hydrocarbon-arsenic composite group: the initial concentration of petroleum hydrocarbon was 4322.30 mg/kg, and Na_2_HAsO_4_ solution and 10 mL of bacterial suspension were added; the inoculum was about 5 × 10^7^ CFU/g soil. The concentration of arsenic was 201.65 mg/kg. Sterilized pure water was added to the control group, and the initial content of petroleum hydrocarbons in the control was 4303.2 mg/kg. pH of the soil in the three groups was 6.8.

The three groups were incubated in a biochemical incubator at 30 °C, and the percentage of water content was maintained at 10% by adding sterilized pure water at regular intervals. The remaining petroleum hydrocarbon content in the soil was measured every 5~7 days.

## 3. Results and Discussion

### 3.1. Metabolic and Taxonomic Characteristics of the Isolates

**Figure 1:**
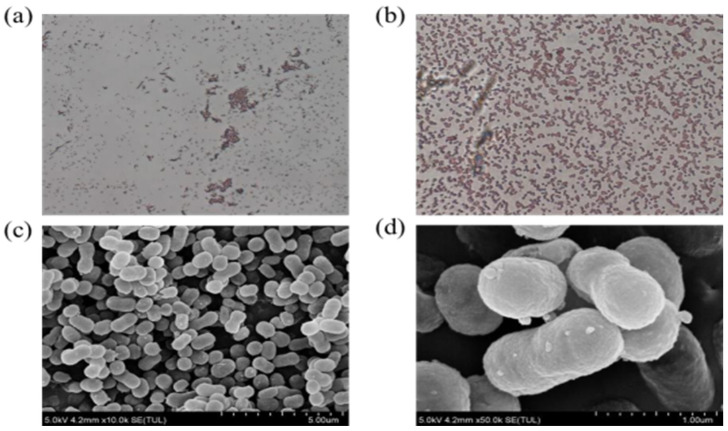
Photographs of cell of strain JYZ-03. (**a**,**b**) Gram stain photo of JYZ-03 (×20 microscopic view; ×100 microscopic view); (**c**,**d**) Scanning electron microscope photo of JYZ-03 (5.00 μm SEM; 1.00 μm SEM).

**Figure 2:**
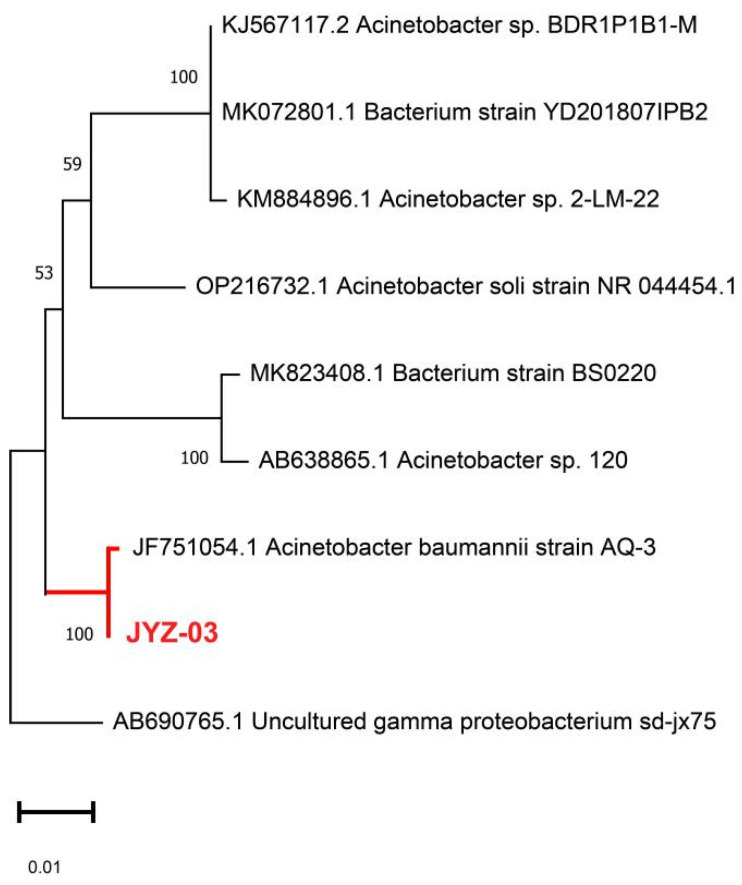
Phylogenetic tree of soil bacterial strains capable of degrading total petroleum hydrocarbons.

Five strains JYZ(01-05) were isolated from soil samples contaminated with petroleum hydrocarbons near a gas station in Wuhan, Hubei Province, China. The objective was to determine whether these strains could form a clear zone on MSM containing diesel as the sole carbon source. After 5 days of culture, the degradation efficiency of JYZ-03 was 64.92%, and the degradation efficiency of the other 4 strains was lower than 50%. Unlike other strains isolated in this experiment, JYZ-03 retained the capacity to degrade petroleum hydrocarbons even under arsenic (As) stress. Gram staining, contact enzyme and amylase reactions, methyl red tests, strain emulsification performance evaluations, and SEM analysis, Strain JYZ-03 was a kind of Gram-negative rod-shaped bacterium (Figure 1), and 16S rDNA gene sequence analysis verified that strain JYZ-03 belonged to *Acinetobacter* sp. (Figure 2). Prior studies showed that *Acinetobacter* sp. are ubiquitous in the environment and have been isolated from oil-polluted samples in which they utilized crude oil as their sole carbon source. *Acinetobacter* sp. is the main bacterial taxon implicated in petroleum hydrocarbon biodegradation [23,24].

### 3.2. Diesel Oil Degradation Assay

Diesel oil is a complex mixture of medium- and long-chain n-alkanes (C11–C25). The bacterial strain alters the alkane composition of the diesel oil it degrades, as shown in Table 2. The chromatogram of the residual diesel oil in MSM revealed the alkanes n-undecanone (C11) to n-pentadecane (C25). The characteristic peak intensity of each n-alkane was significantly reduced compared with the control. We also measured the degradation efficiency of each n-alkane component in the diesel oil at the end of the culture. The most n-alkane degradation efficiency of strain JYZ-03 in oil-containing MSM was in the range of 75–100%, and the n-alkanes degradation efficiency was 84.05%, as shown in Table 2. Hence, strain JYZ-03 effectively degraded the major n-alkanes in diesel oil. It is generally more difficult to biodegrade long-carbon chains than short-carbon chain alkanes [23] We found that the degradation efficiency of n-undecane was 48.78%, which was lower than those of other medium- and long-carbon chain alkanes. Undecane is an intermediate in several serial oxidations involving the hydroxylation, dehydrogenation, and decarboxylation of other medium- and long-carbon chain alkanes. The biodegradation efficiency of n-alkanes depends upon their chain length, physicochemical properties, and the characteristics of the bacterial strains using them as carbon and energy sources.

### 3.3. Carbon Source Utilization

The principal components of diesel oil are C11–C25 normal alkanes. However, they do not represent all petroleum hydrocarbon pollutants. Branched alkanes and polycyclic aromatic hydrocarbons (PAHs) are also important components of petroleum hydrocarbons. Thus, more than 10 carbon sources were measured and tested as potential carbon substrates for strain JYZ-03, including long-chain and branched alkanes as well as polycyclic aromatic hydrocarbons (PAHs). The foregoing chemical classes are the major components of petroleum hydrocarbons and are ubiquitous in contaminated soil. Long-chain alkanes are often more toxic than short-chain alkanes. For this reason, it is equally important to study the microbial degradation efficiency of long-chain normal alkanes. Table 3 shows that strain JYZ-03 reached the highest degradation efficiency in long-chain (C26–C38) n-alkanes in the culture medium with C32.

Several strains of *Acinetobacter* sp. may preferentially degrade C10–C30 alkanes, as these bacteria harbor genes encoding the hydrocarbon-degrading enzymes n-alkane dioxygenase and n-alkane hydroxylase [24,25]. These bacterial enzymes aerobically biodegrade alkanes.

Strain JYZ-03 degrades long-chain alkanes with far lower efficiency than medium-chain alkanes.

In many cases, the toxicity of branched alkanes and PAHs is higher, and their degradation is more difficult than those of linear alkanes. Branched alkanes have complex structures and are sterically hindered. Therefore, it is challenging to identify the enzymes that are capable of degrading them. Branched alkane biodegradation is complex and difficult [22]. The branched alkanes in diesel oil include mainly pristane (2,6,10,14-tetramethylpentadecane) and phytane (2,6,10,14-tetramethylhexadecane). We selected various branched alkanes and PAHs as sole carbon sources to study the ability of strain JYZ-03 to degrade them. Table 3 shows that the pristane and phytane degradation efficiencies of strain JYZ-03 were 63.18% and 70.52%, respectively. Thus, the bacterium could effectively decompose them. PAHs are stable, have unique molecular structures, and their toxicity increases with the number of benzene rings. Microbial action is primarily responsible for removing PAHS from the environment [26]. Naphthalene (NAP) and phenanthrene (PHE) have two and three benzene rings, respectively, and were used as the sole carbon sources for strain JYZ-03 here to determine its capacity to degrade this class of substances. Table 3 shows that in MSM containing 50 mg/L naphthalene or 50 mg/L phenanthrene, the degradation efficiencies were 36.62% and 26.71%, respectively. Strain JYZ-03 could effectively degrade PAHs with two and three benzene rings and use branched alkanes and PAHs as its sole carbon and energy sources. The carbon source utilization experiment partially elucidated the degradation ability of strain JYZ-03 to a different component of petroleum hydrocarbon.

### 3.4. Effects of Environmental Factors on Petroleum Hydrocarbon Removal by JYZ-03

**Figure 3:**
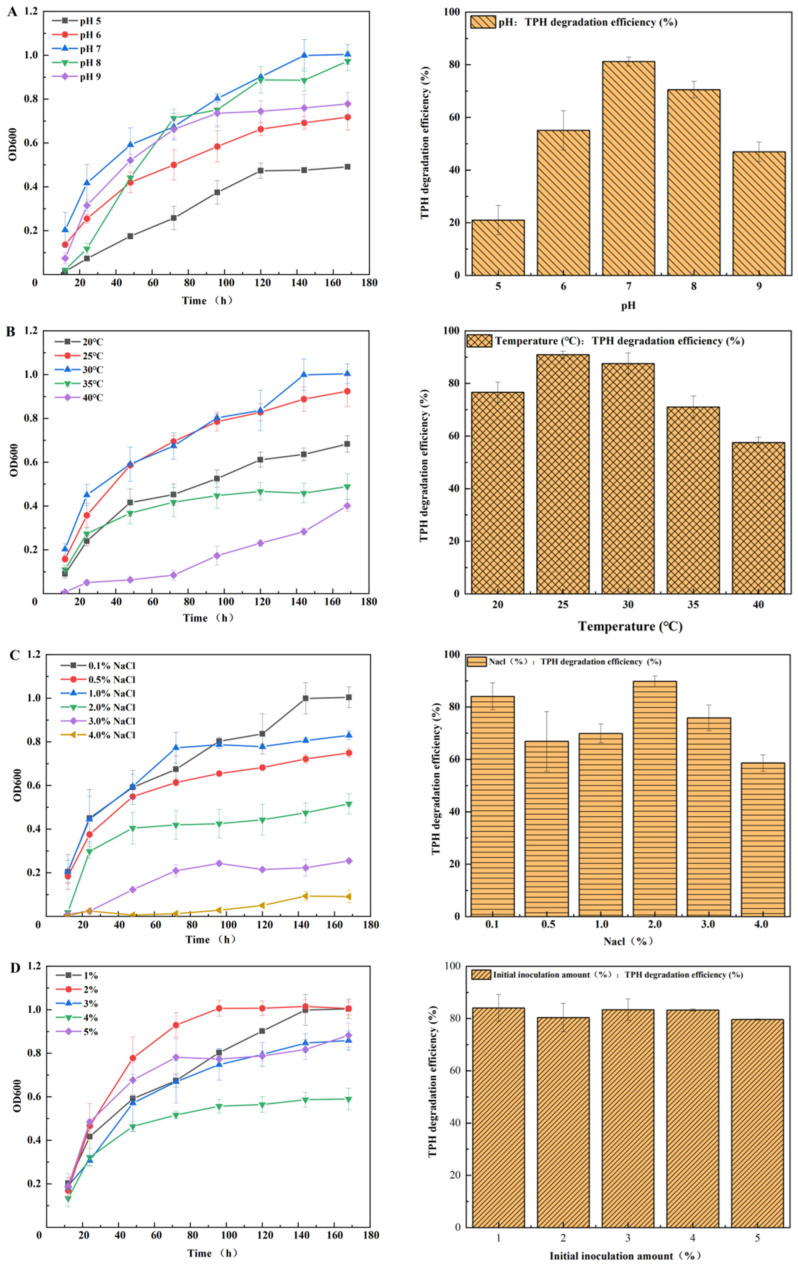
Effect of environmental factors on diesel oil removal by strain JYZ-03. (**A**) pH; (**B**) incubation temperature; (**C**) NaCl concentration (%); (**D**) inoculum concentration. The experiments of “TPH degradation efficiency” were performed in MSM medium, and the experiments of the “Turbidity” (OD_600_) were performed in LB medium.

Several petroleum hydrocarbon degradation tests were conducted to test the effect of different environmental factors such as pH, temperature, salinity, and inoculum size on petroleum hydrocarbon degradation by the JYZ-03 strain. The results are presented in Figure 3. Figure 3A shows that pH directly affected bacterial growth, metabolism, and enzyme activity. Since pH directly affects the growth and metabolism of bacteria and the activity of related degrading enzymes, the pH of the culture medium is one of the key factors affecting the degradation of petroleum hydrocarbons. With the increase in pH value, the degradation efficiency generally increased first and then decreased. When pH was 7, the optimum growth pH of bacteria was obtained, and the degradation efficiency of petroleum hydrocarbon was 81.18%. When pH was 8, the degradation efficiency of petroleum hydrocarbons decreased slightly to 70.51%, indicating that the strain grew faster in neutral and weakly alkaline environments and had a higher degradation efficiency of petroleum hydrocarbons, which may be caused by the long-term maintenance of neutral pH of the medium during the acclimation stage. However, in the weak acidic environment with pH 5–6, the growth rate of bacteria showed a delayed period, and the bacterial density was significantly lower than that in the neutral and weak alkaline environment when it reached the stable stage, which may be related to the sudden change of pH in the medium during acclimation and activation. Degradation efficiency first rose and then fell with increasing pH. Thus, culture medium pH was a key factor affecting petroleum hydrocarbon degradation [27,28,29].

Temperature also plays an important role in petroleum hydrocarbon degradation as it affects both diesel physicochemistry and microbial metabolism. The effects of various culture temperatures on strain JYZ-03 growth rate and degradation efficiency are shown in Figure 3B. Microbial petroleum hydrocarbon degradation efficiency first increased and then decreased with increasing temperature. The optimal culture temperature range for strain JYZ-03 was 25–30 °C. The bacterium had a low tolerance for higher temperatures. *Acinetobacter* are strictly aerobic organisms and use oxygen as the terminal electron acceptor. All strains can grow well between 20 and 30 °C. Most strains have an optimum of 33–35 °C, although some fail to grow at 37 °C. It is recommended that an incubation temperature of 30 °C is used, and in some cases, the use of a lower temperature may be advisable in addition to 30 °C. Verma [30] reported similar results for bacterial degradation of oily sludge.

Salinity directly affects osmotic pressure in bacterial cells. The effects of different NaCl concentrations on strain JYZ-03 growth and degradation efficiency are shown in Figure 3C. Salinity somewhat affected degradation efficiency, possibly because there were other inorganic salts in the culture medium and the degrading bacteria had only limited salinity tolerance [31,32,33]. Zhang et al. also reported that although the salt tolerance of the microbial consortia was not high, the excellent hydrocarbon removal capacity could make the consortia a potential candidate for the bioremediation of petroleum hydrocarbon-contaminated saline–alkaline fields [34,35].

Strain JYZ-03 at initial inoculum concentrations of 1–5% removed to 80% of the diesel oil within 7 days (Figure 3D). The removal of hydrocarbon mainly depends on the capabilities of the microorganisms [36,37,38,39]. Changes in inoculum concentration had no apparent effect on the petroleum hydrocarbon degradation efficiency of strain JYZ-03; that is, very high inoculum concentrations do not necessarily improve petroleum hydrocarbon degradation efficiency. However, the bacterial growth rate has been indeed affected by increasing inoculum size, as the highest turbidity was achieved with 1% and 2%. The higher the inoculum size with the same nutrient content implies the faster the nutrient depletion, so turbidity reaching larger inoculum sizes was lower (Figure 3D, right).

### 3.5. Effects of Heavy Metal Stress on Petroleum Hydrocarbon Removal by JYZ-03

**Figure 4:**
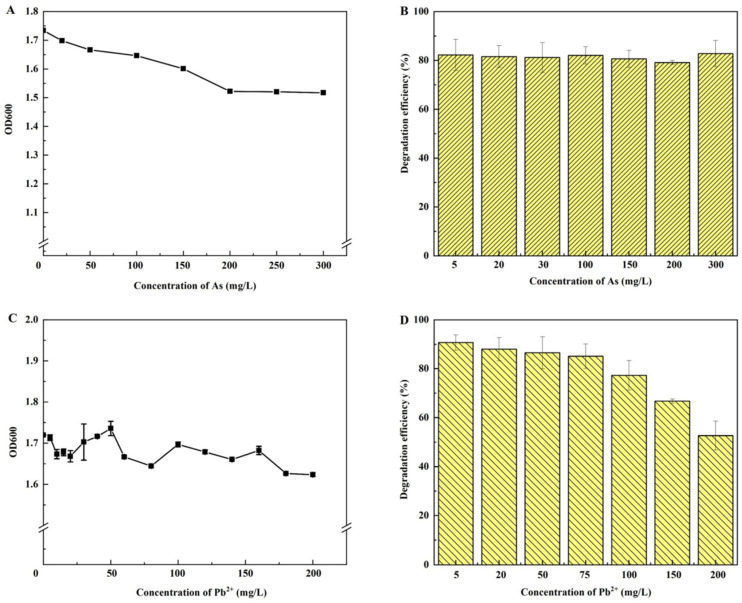
Effects of different heavy metal concentrations on strain JYZ-03 growth and petroleum hydrocarbon degradation. (**A**) Effects of As(V) on strain JYZ-03 growth; (**B**) petroleum hydrocarbon degradation by strain JYZ-03 at various As concentrations; (**C**) effects of Pb^2+^ on strain JYZ-03 growth; (**D**) petroleum hydrocarbon degradation by strain JYZ-03 at various Pb^2+^ concentrations.

Heavy metals are toxic to microorganisms. To determine the toxicity of each heavy metal to strain JYZ-03, we first assessed the effects of As and Pb^2+^ on the growth of strain JYZ-03 by adding various heavy metal concentrations to the LB medium. Figure 4A shows that As(V) markedly and uniquely affected strain JYZ-03 growth. The JYZ-03 biomass remained high even at elevated As(V) concentrations. Hence, strain JYZ-03 has strong tolerance for As(V). The effects of Pb^2+^ on strain JYZ-03 are shown in Figure 4C. At medium-range Pb^2+^ concentrations (0–200 mg/L), the OD_600_ was in the range of 1.61–1.72 after 12 h culture, and the growth of strain JYZ-03 was only slightly affected. Thus, strain JYZ-03 has a certain degree of Pb^2+^ tolerance, at least at Pb^2+^ concentrations tested.

Different heavy metal concentrations were added to MSM to establish their effects on the efficiency of petroleum hydrocarbon degradation by strain JYZ-03. Figure 4B shows that even at 300 mg/L As(V), the petroleum hydrocarbon degradation efficiency of strain JYZ-03 was more than 80%. Furthermore, the petroleum hydrocarbon degradation efficiency of strain JYZ-03 did not apparently decrease with increasing As(V) concentration. Therefore, strain JYZ-03 could potentially be bioremediate sites co-contaminated with As and petroleum hydrocarbons.

In MSM containing Pb^2+^, the petroleum hydrocarbon degradation efficiency of strain JYZ-03 significantly decreased with increasing Pb^2+^ concentration (Figure 4D). At a Pb^2+^ concentration of 200 mg/L, the petroleum hydrocarbon degradation efficiency was only 52.65%. Thus, high Pb^2+^ concentrations strongly inhibit petroleum hydrocarbon degradation by strain JYZ-03.

### 3.6. Evaluation of the Efficiency of TPH Removal from Soil by JYZ03

**Figure 5:**
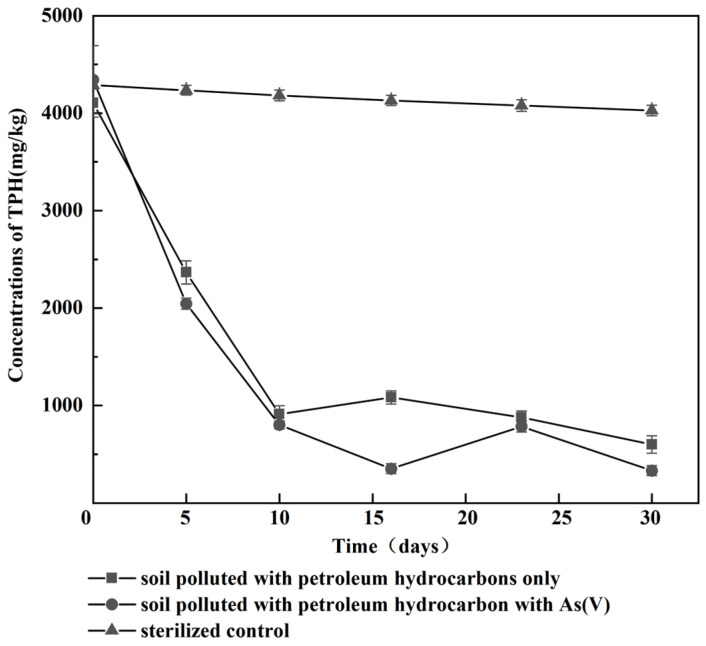
Effect of heavy metals in removal of hydrocarbon-polluted soils by JYZ-03 strain. Closed squares, soil polluted with petroleum hydrocarbons; closed circles, soil polluted with petroleum hydrocarbons plus As(V); closed triangles, sterilized soil polluted without inoculation.

In order to evaluate the degradation capability of the JYZ-03 strain in real polluted soils both in the presence or in the absence of heavy metals, this strain was inoculated in samples of hydrocarbon-polluted soils prepared as described in Section 2.9 of Material and Methods. The results are presented in Figure 5. Figure 5 shows that there was no significant difference in the removal of TPHs from the soil by JYZ03 in the compound-polluted soil of petroleum hydrocarbon with As(V) and single petroleum hydrocarbon-polluted soil. The degradation efficiency increased gradually within 10 days after the biological enhancement treatment, and then, the content of petroleum hydrocarbons in the soil tended to become stable, possibly because during the degradation process, the pollutants are adsorbed by the soil colloidal components, the carbon source required by microorganisms decreases, and the strains gradually decay or are outcompeted by indigenous microorganisms. Therefore, the degradation rate of petroleum hydrocarbons is far lower than that in the initial stage of remediation, as well as reported by Palanisamy [40].

## 4. Conclusions

Heavy metals occur in various forms in petroleum hydrocarbon-contaminated environments. However, certain bacterial strains that utilize petroleum hydrocarbons as carbon and energy sources are also resistant to heavy metals and are, therefore, suitable for application in bioremediation technology. We detected and isolated *Acinetobacter* baumannii strain JYZ-03 from soils contaminated with both petroleum hydrocarbons and heavy metals and established that it could effectively remove the former and had a high tolerance for the latter, including As(V). The petroleum hydrocarbon degradation efficiency of strain JYZ-03 varied with the molecular structure of the contaminant, including carbon chain length, degree of branching, and number of carbon rings present. The efficiencies with which strain JYZ-03 degraded the medium carbon chain length (C11–C25) and long carbon chain length (C26–C38) n-alkanes in diesel oil were 75–100% and 3.11–17.55%, respectively. It degraded branched alkanes and phytane by 63.18% and 70.52%, respectively, and the PAHs naphthalene and phenanthrene by 36.62% and 26.71%, respectively. It also degraded >76.97% of the diesel oil under physicochemical conditions optimal for its growth and performance, namely, pH 7, 25–30 °C, and 0.1–1% salinity. Petroleum hydrocarbon degradation by strain JYZ-03 first increased and then decreased with increasing pH. Strain JYZ-03 grew best in neutral and weakly alkaline environments. Its petroleum hydrocarbon degradation efficiency first increased and then decreased with increasing temperature. Strain JYZ-03 grew best at 25–30 °C. It had high salt tolerance, and even 3.0% (*w*/*v*) NaCl did not inhibit its growth. Within a certain range, the initial inoculum size did not markedly affect petroleum hydrocarbon degradation. The present study demonstrated that strain JYZ-03 effectively bioremediates petroleum hydrocarbon-contaminated soil even in the presence of As stress.

## Figures and Tables

**Table 1:** Basic soil physicochemical properties.

Soil Property	Value
pH	8.24
Soil organic matter (SOM) (mg/kg)	78,650
Total N (mg/kg)	3600
Total P (mg/kg)	760
As (mg/kg)	8.6
Total petroleum hydrocarbons (TPH) (mg/kg)	318.88

**Table 2:** Residual n-alkanes concentrations of 1% diesel oil in MSM medium for 7 days.

N-Alkanes	Initial Concentrations (mg·L^−1^)	Final Concentrations (mg·L^−1^)	Degradation Rate (%)
C11	11.94 ± 0.80	6.12 ± 0.01	48.78 ± 0.97
C12	113.70 ± 5.87	28.82 ± 0.3.6	74.65 ± 6.05
C13	294.27 ± 18.76	36.75 ± 0.82	87.51 ± 4.36
C14	644.28 ± 43.60	120.80 ± 3.03	81.25 ± 6.95
C15	794.74 ± 54.45	128.35 ± 2.78	83.85 ± 5.10
C16	596.48 ± 40.25	93.47 ± 1.78	84.33 ± 4.42
C17	355.69 ± 25.30	52.64 ± 0.89	85.20 ± 3.41
C18	360.10 ± 12.02	57.51 ± 0.61	84.03 ± 5.09
C19	346.46 ± 22.48	48.95 ± 0.86	85.87 ± 3.95
C20	235.60 ± 18.70	37.84 ± 1.44	83.94 ± 7.68
C21	202.03 ± 14.08	21.64 ± 0.54	89.29 ± 3.85
C22	114.92 ± 9.14	12.43 ± 0.33	89.18 ± 3.59
C23	47.27 ± 3.43	10.07 ± 0.29	78.70 ± 8.59
C24	26.43 ± 2.30	1.68 ± 0.21	93.65 ± 8.98
C25	13.14 ± 0.80	0.00 ± 0.00	100.00 ± 0.00
Average	4157.00 ± 246.00	663.04 ± 12.57	84.05 ± 5.11

**Table 3:** Bacterial degradation efficiency of various carbon sources.

Carbon Sources	Degradation Rate (%)
C26	17.56 ± 0.04
C28	18.56 ± 1.77
C30	9.94 ± 2.72
C32	25.88 ± 3.44
C34	20.24 ± 1.32
C36	11.16 ± 1.42
C38	3.11 ± 1.90
Pristane	63.18 ± 9.06
Phytane	70.52 ± 8.58
Naphthalene	36.62 ± 2.78
Phenanthrene	26.71 ± 4.46

## Data Availability

Not applicable.
